# Studies on 1,4-Quinone Derivatives Exhibiting Anti-Leukemic Activity along with Anti-Colorectal and Anti-Breast Cancer Effects

**DOI:** 10.3390/molecules28010077

**Published:** 2022-12-22

**Authors:** Halilibrahim Ciftci, Belgin Sever, Nusret Kaya, Nilüfer Bayrak, Mahmut Yıldız, Hatice Yıldırım, Hiroshi Tateishi, Masami Otsuka, Mikako Fujita, Amaç Fatih TuYuN

**Affiliations:** 1 Department of Drug Discovery, Science Farm Ltd., Kumamoto 862-0976, Japan; 2 Medicinal and Biological Chemistry Science Farm Joint Research Laboratory, Faculty of Life Sciences, Kumamoto University, Kumamoto 862-0973, Japan; 3 Department of Molecular Biology and Genetics, Koc University, Istanbul 34450, Turkey; 4 Department of Pharmaceutical Chemistry, Faculty of Pharmacy, Anadolu University, Eskisehir 26470, Turkey; 5 Metallurgical and Materials Department, Izmir Katip Celebi University, Izmir 35620, Turkey; 6 Department of Chemistry, Faculty of Science, Istanbul University, Fatih, Istanbul 34126, Turkey; 7 Chemistry Department, Gebze Technical University, Gebze, Kocaeli 41400, Turkey; 8 Department of Chemistry, Faculty of Engineering, Istanbul University-Cerrahpasa, Avcılar, Istanbul 34320, Turkey

**Keywords:** CML, CRC, breast cancer, quinone, apoptosis, DNA binding potential, molecular docking, drug-likeness

## Abstract

Colorectal cancer (CRC), breast cancer, and chronic myeloid leukemia (CML) are life-threatening malignancies worldwide. Although potent therapeutic and screening strategies have been developed so far, these cancer types are still major public health problems. Therefore, the exploration of more potent and selective new agents is urgently required for the treatment of these cancers. Quinones represent one of the most important structures in anticancer drug discovery. We have previously identified a series of quinone-based compounds (**ABQ-1-17**) as anti-CML agents. In the current work, **ABQ-3** was taken to the National Cancer Institute (NCI) for screening to determine its in vitro antiproliferative effects against a large panel of human tumor cell lines at five doses. **ABQ-3** revealed significant growth inhibition against HCT-116 CRC and MCF-7 breast cancer cells with 2.00 µM and 2.35 µM GI_50_ values, respectively. The MTT test also showed that **ABQ-3** possessed anticancer effects towards HCT-116 and MCF-7 cells with IC_50_ values of 5.22 ± 2.41 μM and 7.46 ± 2.76 μM, respectively. Further experiments indicated that **ABQ-3** induced apoptosis in both cell lines, and molecular docking studies explicitly suggested that **ABQ-3** exhibited DNA binding in a similar fashion to previously reported compounds. Based on in silico pharmacokinetic prediction, **ABQ-3** might display drug-like features enabling this compound to become a lead molecule for future studies.

## 1. Introduction

Colorectal cancer (CRC) is a very malignant and prevalent tumor worldwide. Age, local inflammatory changes, genetic components, and a substantial number of environmental and lifestyle factors account for important risks of CRC development. The great majority of patients with CRC are generally diagnosed at late stages with metastases requiring the administration of radiation therapy and chemotherapy as the leading therapeutic strategies for controlling the disease. Although these therapeutic options have evolved to targeted therapy and immunotherapy, the prognosis of CRC has never reached to the desired rate beyond a delay of disease progression so far. The complexity of the underlying mechanisms of the disease and the development of resistance are the major challenges for the treatment failure [1,2,3,4,5,6,7].

On the other hand, breast cancer is the most prevalent and second most deadly cancer among women across the world. It is heterogenous in nature, containing various subtypes with specific properties. Age, heredity, and steroid hormones are major risk factors in breast cancer development. In spite of traditional treatment strategies, including surgery, chemotherapy, and radiotherapy along with hormonotherapy and targeted therapy options to improve overall survival in breast cancer patients, drug resistance is the major impediment to achieving effective anticancer treatment [8,9,10,11,12].

Chronic myeloid leukemia (CML) is a myeloproliferative disease characterized by the presence of chromosomal abnormality leading to the aberrant Abelson oncogene (Abl)-breakpoint cluster region (Bcr) gene fusion, which encodes for the Bcr-Abl tyrosine kinase. Tyrosine kinase inhibitors such as imatinib, dasatinib, nilotinib, bosutinib, and ponatinib are the mainstay of CML treatment. However, due to the resistance and toxicity problems emerging during treatment with these agents, new treatment approaches to be efficient in CML treatment are required [13,14,15].

Apoptosis, known as programmed cell death, maintains the elimination of large numbers of cells that are no longer needed without causing any harm to the developing organism [16,17]. Necrosis, which is the death of cells in alive tissue, triggers inflammation, whereas apoptosis does not. [18,19]. In recent years, the major goal of clinical oncologists has been to develop new treatment options that support the effective elimination of cancer cells through apoptosis. Several signaling pathways are activated by cellular stress, DNA damage, and immune surveillance to mediate apoptosis [20].

It has also gained great importance in developing new, more effective, and selective drugs that precisely target/block the changes that cause cancer growth and proliferation. Natural products are associated with the development of novel therapeutic products for cancer treatment; new natural product analogs revealing great chemical diversity and high potential biological activities have become the apple of the eye of scientists [21,22,23,24,25,26].

**Figure 1:**
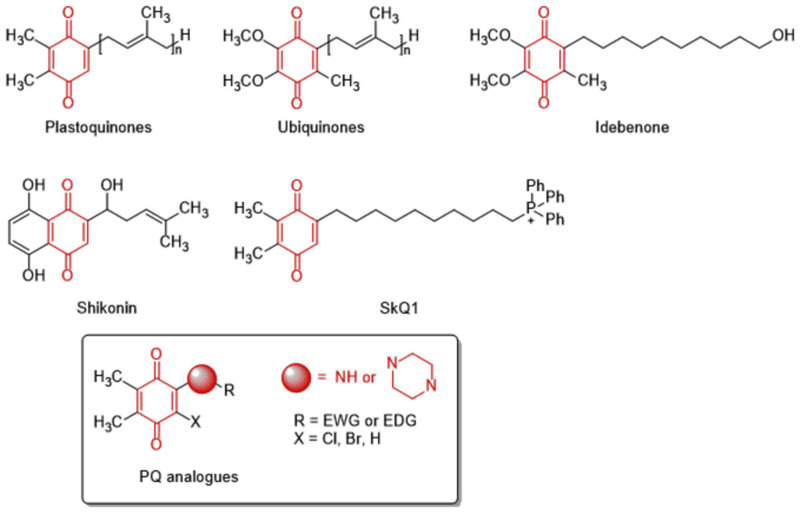
The structures of natural and synthesized substances containing 1,4-quinone moiety.

**Figure 2:**
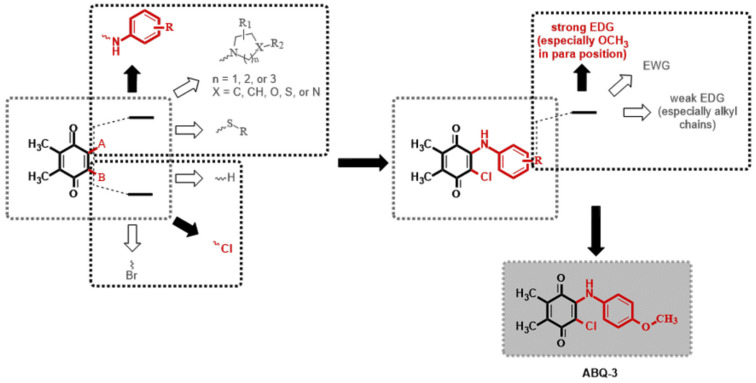
Possible modifications of dimethyl-1,4-benzoquinone moiety within our previous studies and the selection of a target analog (Both black filled arrow and red colored moiety are used to indicate the selected structure in this study. On the other hand, a white filled arrow and dark grey colored moiety are used to show other possible moieties not included in this study).

The 1,4-quinone moiety is a prominent source of biologically active substances, as shown in Figure 1 [27,28,29,30]. 1,4-Quinones have attracted a great deal of attention due to their diverse biological activities, including anticancer, antioxidant, antiviral, antibacterial, and antifungal [31,32,33,34]. Many synthetic amino substituted 1,4-quinone molecules comprising 1,4-quinone and amino moiety have been discovered for the development of novel medications with anticancer properties [35,36]. Despite the great advantages of quinone in medicinal chemistry, there are some concerns as to its use as an anticancer agent; this includes its potential with covalent binding to microsomal proteins and also to DNA along with an increase in the formation of reactive oxygen species [37,38]. Therefore, a new quinone-based anticancer drug design must be developed considering every aspect. We have previously discovered a new class of plastoquinone, which hereinafter has been referred to as PQ analogs comprising 2,3-dimethyl-1,4-benzoquinone and amino substitutions as antileukemic agents and have identified some of them as lead molecules with in vitro and in silico studies [23,24,25,26]. Concerning all PQ analogs, we have come out with three main groups in modification named halogenated (brominated and chlorinated) and nonhalogenated analogs. Additionally, varieties of the substituent, such as electron-withdrawing group(s) (EWG) or electron-donating group(s) (EDG) within amino moiety were also designed, obtained, and evaluated by our group for their anticancer effects. Of these main groups, biological experiments asserted that the chlorinated PQ analogs exhibited potent inhibition against studied cancer cell lines and, thus, were selected to be the most potent group. Specifically, these analogs containing the mono alkoxy group (methoxy and ethoxy) at a *para* position revealed the highest inhibitory capacity. The biological potency decreased in these structures with the extension of the bulky alkyl tail in the alkoxy group (butoxy, hexyloxy, and octyloxy), illustrated in Figure 2 [23].

In order to enhance biological activity, we additionally studied the lead molecules to determine further activity against HCT-116 CRC and MCF7 breast cancer cells. Moreover, the most effective anticancer compound was subjected to further mechanistic experiments, such as the in vitro determination of levels of apoptosis in both cell lines and the in silico analysis of the DNA binding mode. Several pharmacokinetic determinants of this compound were also anticipated in silico.

## 2. Results

### 2.1. Biological Activity

#### 2.1.1. *In Vitro* Anticancer Screening

**Figure 3:**
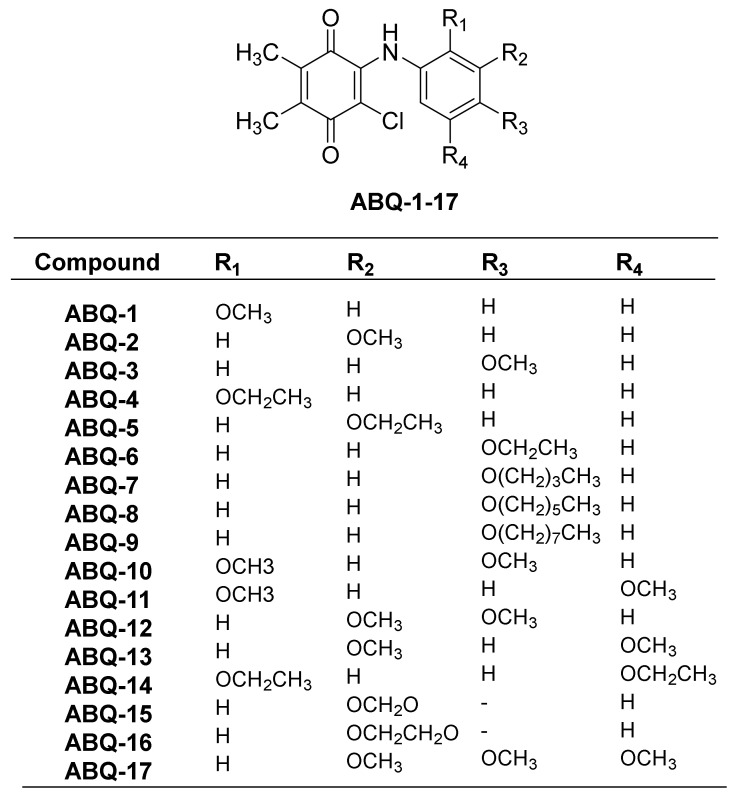
General chemical structure of **ABQ-1-17** along with substitutions.

In our previous study, PQ analogs (Figure 3) were documented to show anticancer activities against leukemia cell lines [23]. This was followed by other reports claiming the anticancer potential of PQ analogs against breast and colon cancers as evidenced by their cytotoxic activities [22,39,40]. In the current work, the NCI initially screened three PQ analogs (**ABQ-3**, NCI: D-827196/1, **ABQ-11**, NCI: D-827197/1, and **ABQ-12**, NCI: D-827198/1) towards 60 cancer cell lines [41] at a single dose concentration (10 µM) on different cancer types, namely CRC, breast cancer, leukemia, melanoma, central nervous system (CNS), non-small cell lung cancer (NSCLC), ovarian, and renal and prostate cancer cell lines [42,43]. After this evaluation, **ABQ-3** (2-chloro-3-((4-methoxyphenyl)amino)-5,6-dimethyl-1,4-benzoquinone) was appointed as a lead PQ analog because of its significant selective anticancer potential compared to **ABQ-11** and **ABQ-12** for five-dose in vitro anticancer activity assessment in the range of 0.01–100 µM.

In the current work, GI_50_ (growth inhibitory activity), TGI (cytostatic activity), and LC_50_ (cytotoxic activity)) [44] were used to evaluate the biological potential of the selected PQ analog **ABQ-3**. The GI_50_ is an indicative concentration with a 50% growth inhibition, whereas TGI refers to the total growth inhibitory activity, and LC_50_ is an indicative concentration in which 50% of cancer cells died. These parameters were calculated for each cell line from the log concentration versus % growth inhibition curves on 60 human cancer cell lines to create dose–response curves [43,45].

The selected PQ analog **ABQ-3** showed high anticancer effects against all leukemia cell lines with GI_50_ values of around 2.50 µM. **ABQ-3** revealed sensitivity towards all leukemia cell lines in concordance with our previous encouraging results that the IC_50_ values of **ABQ-3** against K562, Jurkat, and MT-2 cells were found as 0.82 ± 0.07 µM, 1.51 ± 0.29 µM and 5.41 ± 0.95 µM, respectively. **ABQ-3** also exerted low cytotoxicity against the healthy cell line (Table 1).

**Figure 4:**
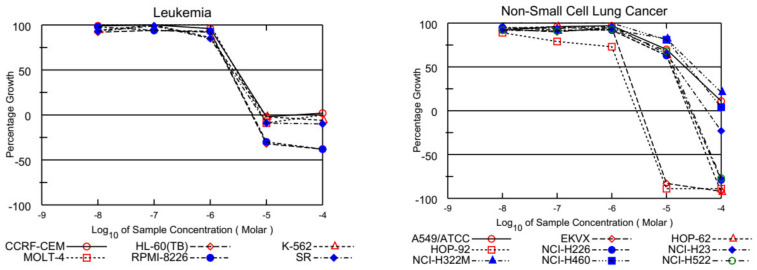

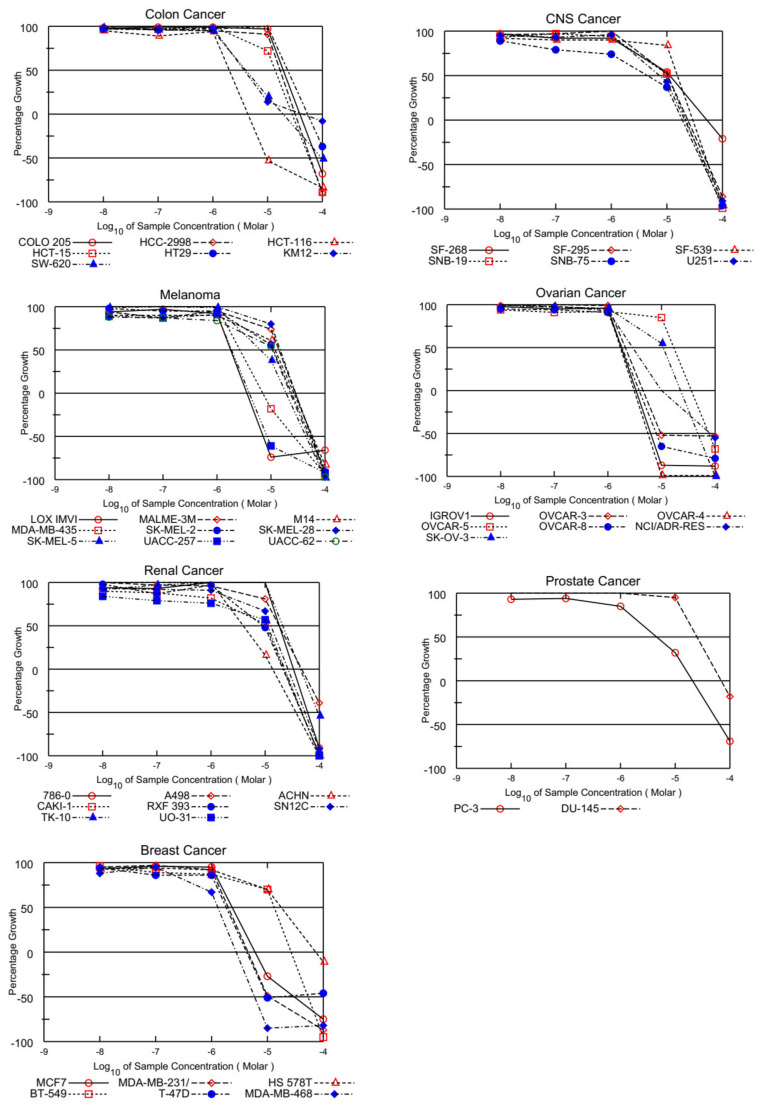
Graphical presentation of growth inhibition of **ABQ-3** at five-dose concentrations.

On the other hand, EKVX, HOP-92 (NSCLC), HCT-116, SW-620 (CRC), LOX IMVI, MDA-MB-435, and UACC-257 (melanoma), IGROV1, OVCAR-3, OVCAR-4, OVCAR-8, and NCI/ADR-RES (ovarian cancer), MCF-7, MDA-MB-231/ATCC, T-47D, and MDA-MB-468 (breast cancer) were also found susceptible to **ABQ-3** (Table 2). This analog displayed excellent cytotoxicity towards HL-60, K-562, RPMI-8226, and SR cells with TGI ranging from 5.54 to 9.59 µM. In addition, LC_50_ values were higher than 100 µM against the entire panel of leukemia cells. Other significant results were recorded against EKVX and HOP-92 (NSCLC) with the values of 1.79, and 1.40 µM GI_50_, respectively. HCT-116 (CRC) had a value of 2.00 µM GI_50_, LOX IMVI, and UACC-257 (melanoma) had values of 1.79 and 1.86 µM GI_50_, IGROV1, OVCAR-3, OVCAR-4, OVCAR-8, and NCI/ADR-RES (ovarian cancer) had GI_50_ values in the range of 1.71–2.12 µM, and MCF-7, MDA-MB-231/ATCC, T-47D, and MDA-MB-468 (breast cancer) had GI_50_ values in the range of 1.29–2.35 µM. TGI values lower than 20.00 µM were determined with most of the mentioned cell types. Finally, **ABQ-3** demonstrated a potent lethal function towards the majority of cancer cell lines in the range of 5.60–26.60 µM LC_50_ values. All the dose–response curves of **ABQ-3** against the 60 human cancer cell lines are outlined in Figure 4 and Table 2.

#### 2.1.2. MTT Assay on HCT-116 and MCF-7 Cells

**ABQ-3** displayed significant sensitivity towards HCT-116 and MCF-7 cell lines based on NCI GI_50_, TGI, and LC_50_ parameters. In addition, CRC and breast cancer are important research platforms that we have been working on for a long time. Therefore, we further examined the anticancer effects of **ABQ-3** on these two cell lines via an MTT (3-(4,5-dimethyl-2-thiazolyl)-2,5-diphenyltetrazolium bromide) assay at five dose concentrations (1, 3, 10, 30, and 100 μM) in comparison with cisplatin. Cisplatin was selected as a control because cisplatin has been used for the treatment of various cancers such as CRC and breast cancer [46,47,48,49,50].

**Figure 5:**
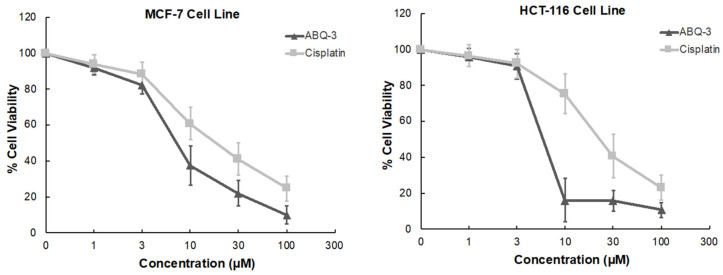
The cytotoxicity of **ABQ-3** at varying concentrations on breast cancer and CRC cells compared to cisplatin.

According to the results, **ABQ-3** inhibited the cell viability of HCT-116 and MCF-7 with notable IC_50_ values of 5.22 ± 2.41 μM and 7.46 ± 2.76 μM compared to cisplatin (IC_50_ = 22.19 ± 5.29 μM: HCT-116 cells; 17.65 ± 4.55 μM: MCF-7 cells) (Table 3). The percentage of viable cells was diminished sharper in HCT-116 cells in comparison to MCF-7 cells between 3 and 10 μM after **ABQ-3** treatment (Figure 5).

#### 2.1.3. Cell Death Investigation

**Figure 6:**
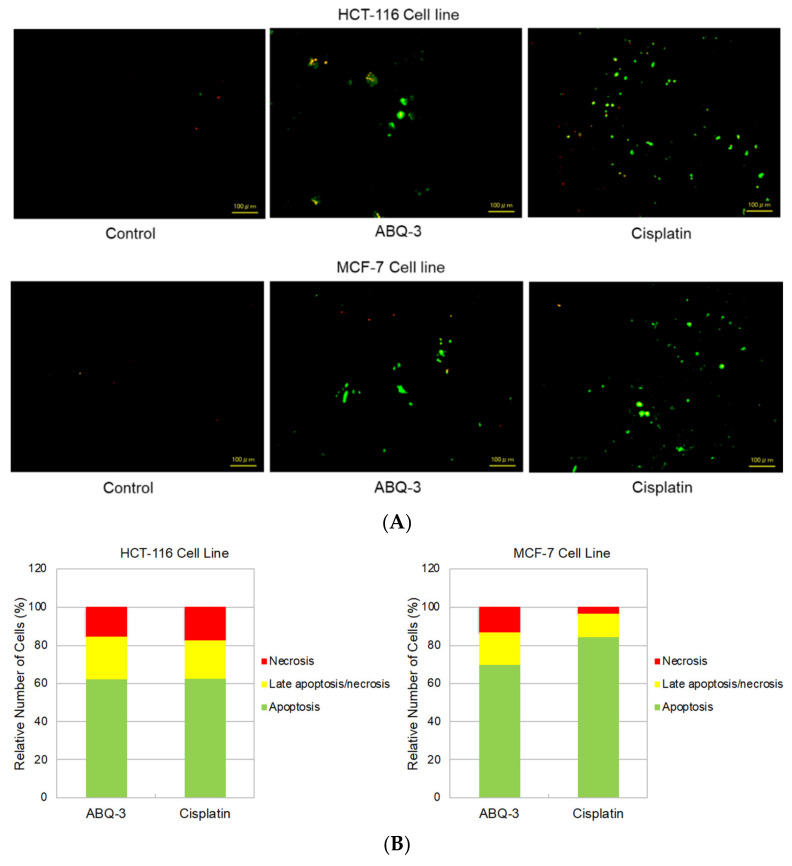

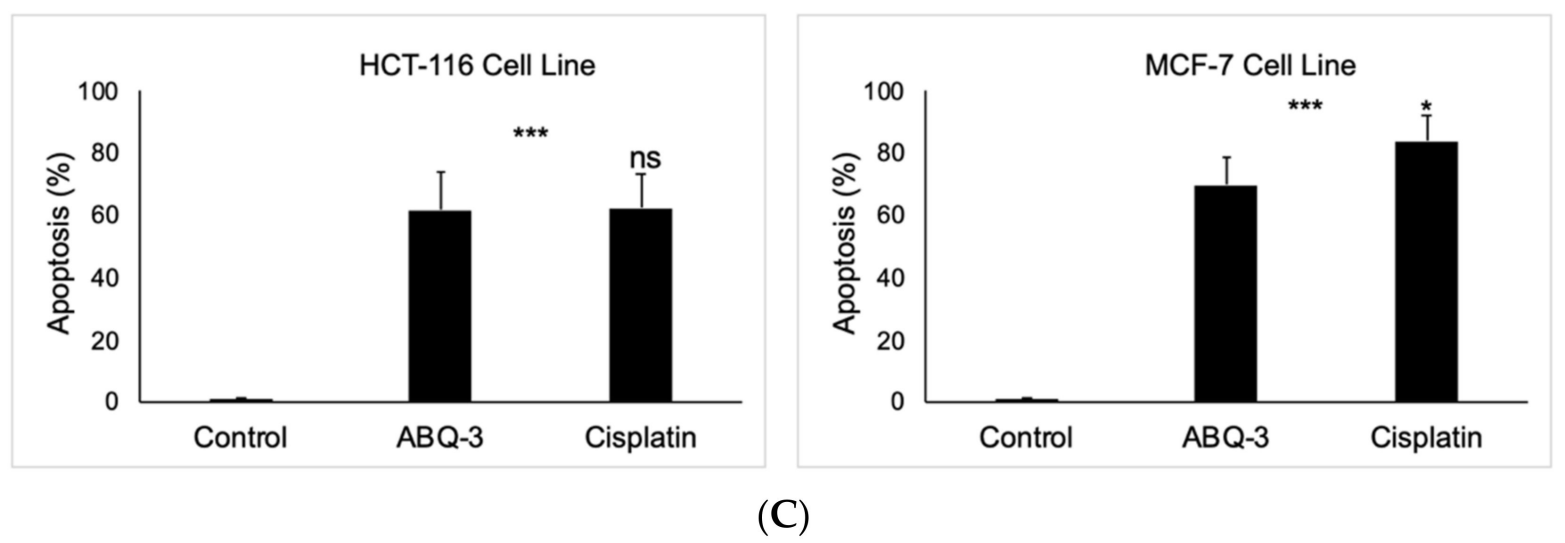
The changes that appeared in HCT-116 and MCF-7 cells after treatment with the control, **ABQ-3**, and cisplatin (**A**) for 12 h. (**B**) A total of 100 randomly selected stained cells were analyzed in each experiment to define the percentage of apoptotic (green), late apoptotic/necrotic (yellow), and necrotic (red) cells (**C**) ***: denotes significant difference from control at *p* < 0.001, *: denotes significant difference from **ABQ-3** at *p* < 0.05, ns: denotes no significant difference between **ABQ-3** and cisplatin. The effect of the control (solvent) is below 1%, and this effect is excluded from the effect of molecules (**ABQ-3** and cisplatin) in (**B**) but is shown in (**C**).

Since **ABQ-3** displayed notable anticancer effects on CRC and breast cancer cells, we further searched for its apoptotic effects in HCT-116 and MCF-7 cells using the annexin V/ethidium homodimer III staining assay, which was detected by a fluorescence microscope representing apoptosis, necrosis, or late apoptosis/necrosis with green, yellow and red staining, respectively (Figure 6A). **ABQ-3** was found to possess similar apoptotic behavior in HCT-116 cells (61.80%) with cisplatin (62.30%). In addition, **ABQ-3** showed 22.70% late apoptotic/necrotic and 15.50% necrotic activity in HCT-116 cells when compared with cisplatin (20.20% and 17.50%, respectively) (Figure 6B). The difference in apoptosis enhancement between **ABQ-3** and cisplatin treatment in HCT-116 cells was found to not be significant; conversely, it was found to be significant in MCF-7 cells, as shown in Figure 6C. MCF-7 cells underwent apoptosis with a higher percentage compared to HCT-116 cells (69.70%) after **ABQ-3** exposure. **ABQ-3** led to 16.67% late apoptosis/necrosis and 13.60% necrosis in MCF-7 cells (Figure 6B).

### 2.2. In Silico Assessment

#### 2.2.1. Molecular Docking

**Figure 7:**
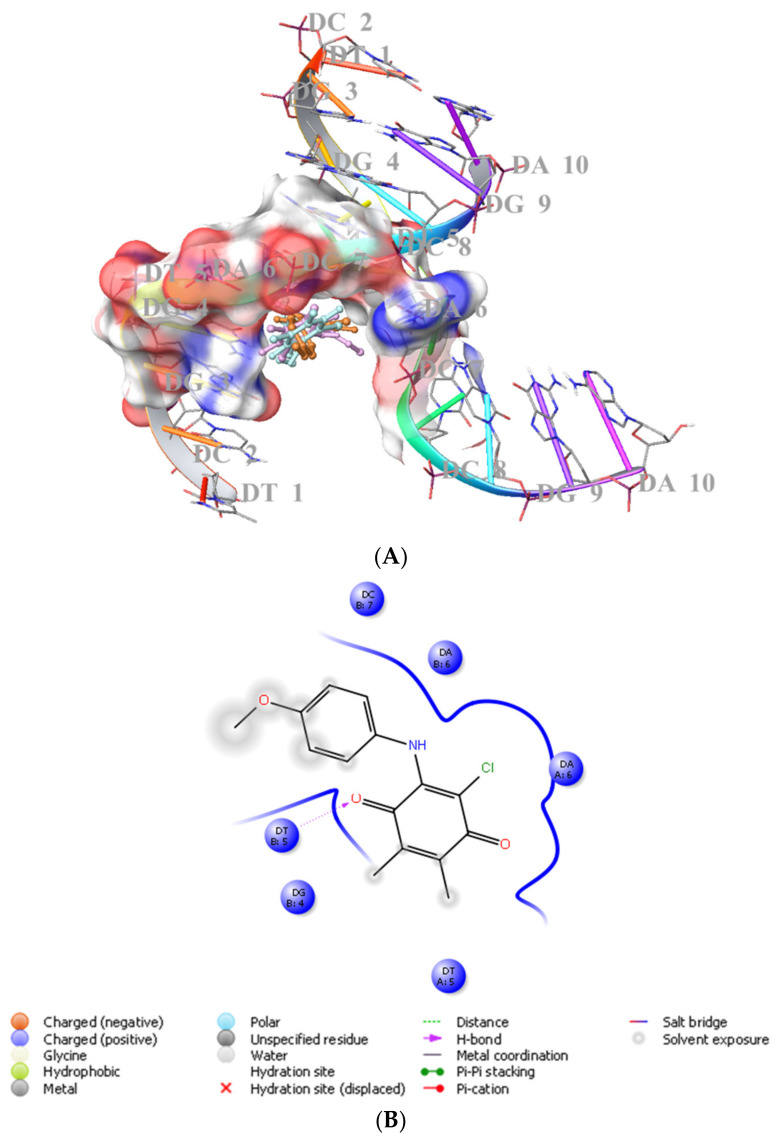
(**A**) Docking poses of **ABQ-3**, **ABQ-11**, and **ABQ-12** and (**B**) docking interactions of **ABQ-3** in the minor groove of DNA (PDB ID: 2GWA) (**ABQ-3**, **ABQ-11**, and **ABQ-12** are colored in orange, plum, and turquoise, respectively).

**Figure 8:**
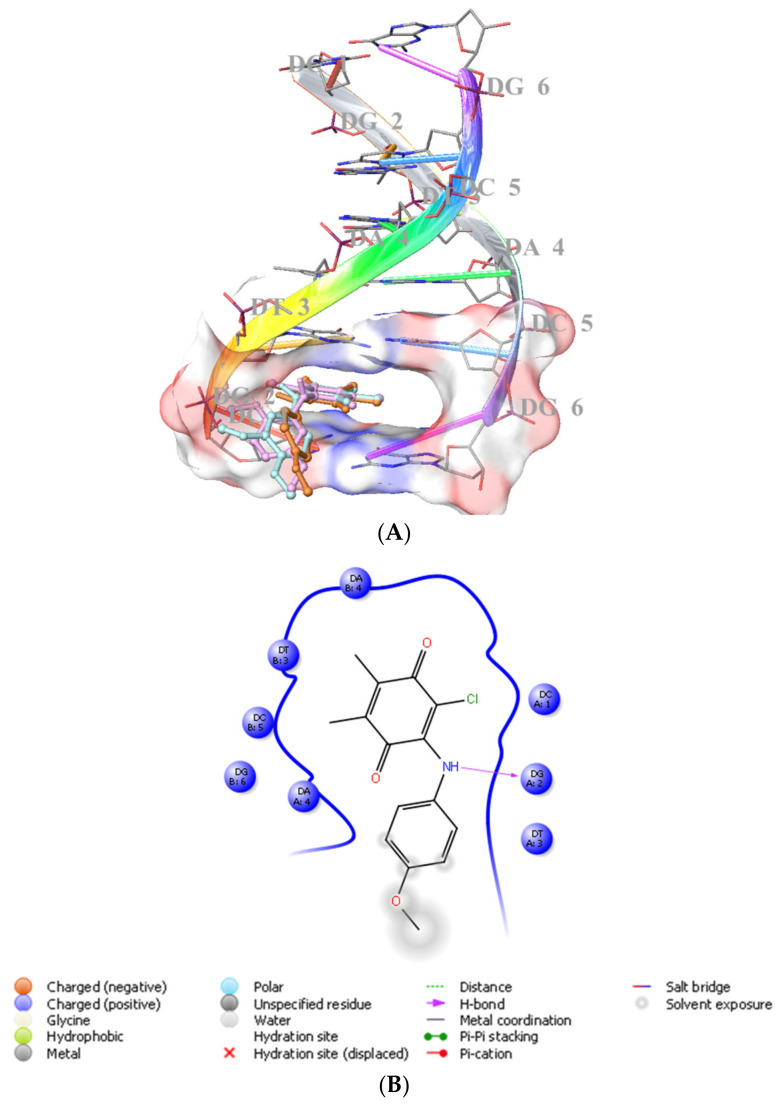
(**A**) Docking poses of **ABQ-3**, **ABQ-11**, and **ABQ-12** and (**B**) docking interactions of **ABQ-3** in the minor groove of DNA (PDB ID: 2GB9) (**ABQ-3**, **ABQ-11**, and **ABQ-12** are colored in orange, plum, and turquoise, respectively).

We previously showed that PQ analogs were capable of binding DNA [40,51,52,53]. Therefore, we also assessed the molecular docking for **ABQ-3** in comparison with **ABQ-11** and **ABQ-12** in the minor groove of DNA (PDB IDs: 2GWA [54] and 2GB9 [55]) using Maestro [56]. The docking scores of **ABQ-3** as −4.980 kcal/mol (PDB ID: 2GWA) and −6.610 kcal/mol (PDB ID: 2GB9) indicated that **ABQ-3** was able to bind to DNA with a higher binding potential compared to **ABQ-11** and **ABQ-12** (Table 4). **ABQ-3** presented hydrogen bonding with DT-5 and DG-2 through the quinone and amino moieties, respectively, in the binding site of DNA (PDB IDs: 2GWA and 2GB9, respectively) (Figure 7A,B and Figure 8A,B).

#### 2.2.2. The Conjecture of Pharmacokinetic Determinants

The pharmacokinetic features of **ABQ-3** were estimated by projecting compounds on the QikProp algorithm [57]. Moreover, the inhibitory effects of **ABQ-3** on cytochrome P450 (CYP) enzymes, the bioavailability, passive gastrointestinal absorption, and brain penetration of **ABQ-3** were in silico anticipated with the help of the SwissADME web service [58,59]. **ABQ-3** served a drug-like character that crucial pharmacokinetic determinants, including the octanol/water partition coefficient (QPlogPo/w), aqueous solubility (QPlogS), human serum albumin binding (QPlogKhsa), and brain/blood partition coefficient (QPlogBB) were coherent within specified ranges (Table 5). **ABQ-3** exhibited an excellent % of absorption (100%) and followed Lipinski’s rule of five and Jorgensen’s rule of three without any violation.

**Figure 9:**
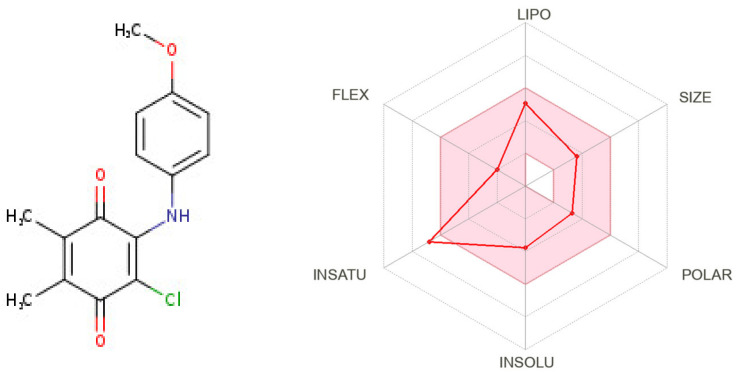
Radar diagrammatic representation of physicochemical properties of **ABQ-3** from SwissADME (Swiss Institute of Bioinformatics, Lausanne, Switzerland).

**Figure 10:**
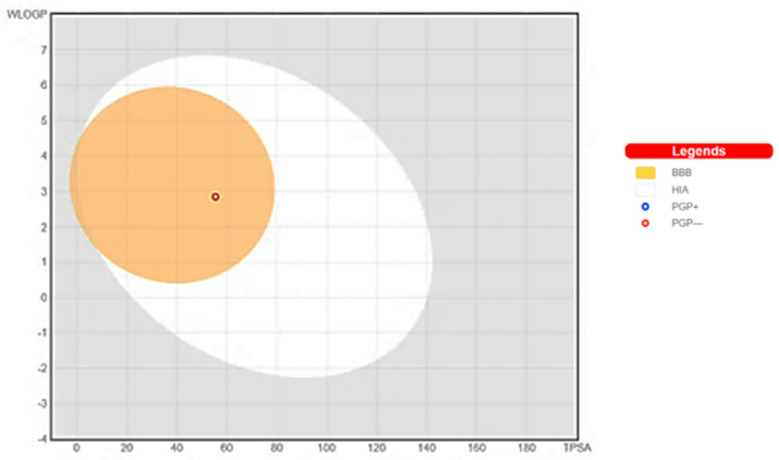
BOILED-Egg representation of **ABQ-3** from SwissADME (Swiss Institute of Bioinformatics, Lausanne, Switzerland).

The demonstration of physicochemical determinants of **ABQ-3** based on the SwissADME tool (Figure 9) indicated the components of the pink area, including saturation, size, polarity, solubility, lipophilicity, and flexibility abbreviated as INSATU, SIZE, POLAR, INSOLU, LIPO, and FLEX, respectively. The red hexagonal line must be entirely in this area for the optimal pharmacokinetic profile. This line of **ABQ-3** was involved in this pink area just out of INSATU with a little range. This compound was able to inhibit CYP1A2, CYP2C19, CYP2C9, and CYP3A4 but matched no CYP2D6 inhibition. The BOILED-egg model (Figure 10) refers to the potential of a compound for passive gastrointestinal absorption and BBB permeation. **ABQ-3** was observed in the egg yolk (yellow region), implying its high penetration through the BBB. **ABQ-3** was also detected with a red dot, explaining that it was not a substrate for P-glycoprotein [59,60,61].

## 3. Discussion

There are many gaps to fill in the treatment of CRC and breast cancer, albeit to the advancements in the screening programs and in the therapeutic options related to prognostic biomarkers. The discovery of new potential anticancer agents to be effective in both cancers may hold the key to the enhancement of treatment responses for patients with CRC and breast cancer. Several lines of evidence have documented that quinone-based compounds, in particular PQ analogs, stand out as promising candidates for anticancer drug discovery [62,63].

**Figure 11:**
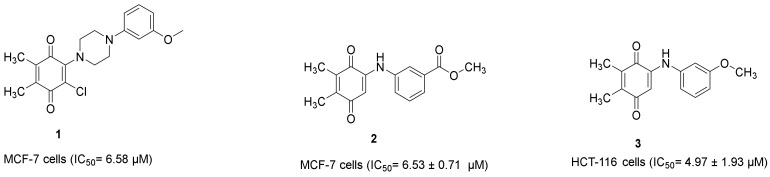
Our research group recently identified PQs to be endowed with anticancer properties against HCT-116 and MCF-7 cells.

Our research group also demonstrated that PQ analogs were endowed with anti-CRC and/or anti-breast cancer properties. Compound **1** (Figure 11) [22] and compound **2** (Figure 11) [39] showed anti-breast cancer effects against the MCF-7 cell line with IC_50_ values of 6.58 μM and 6.53 ± 0.71 μM, respectively, whereas compound **3** (Figure 11) [40] revealed anti-CRC effects towards HCT-116 cells with an IC_50_ value of 4.97 ± 1.93 μM.

In the recent study, **ABQ-3** was selected regarding the protocol of NCI for the evaluation of its antiproliferative effects against a broad range of cancer cell types, including HCT-116 and MCF-7 cells, at five doses. **ABQ-3** exerted notable anticancer effects on HCT-116 and MCF-7 cells with significant GI_50_, TGI, and LC_50_ values. This finding points out that *p*-methoxy substitution on the anilino ring contributed to the anticancer effects of **ABQ-3**. When compared with our aforementioned studies [22,39,40], **ABQ-3** revealed a similar anticancer potential against both cell lines.

Abnormalities in apoptosis can also deteriorate the pathogenesis of CRC and breast cancer and decrease the treatment success causing resistance to current therapy options [64,65,66,67,68,69,70,71]. **ABQ-3** induced apoptosis in both cell lines compared to cisplatin. Comparing our previous results, it was also concluded that **ABQ-3** displayed a similar apoptotic pattern with compound **3** (Figure 10) in HCT-116 cells [40].

Our research group previously reported the DNA binding potential of PQ analogs [40,51,52,53] in the minor groove of DNA (PDB IDs: 2GWA [54] and 2GB9 [55]). **ABQ-3** presented high affinity with a significant docking score value with important hydrogen bonding through quinone moiety. The 4-methoxy moiety made no contribution to the docking interactions of **ABQ-3**.

The determination of the theoretical prediction of the physicochemical properties of a drug candidate has enormously affected successful drug discovery. Several absorptions, distribution, metabolism, and excretion (ADME) parameters of **ABQ-3** were estimated. Based on these parameters, **ABQ-3** was determined to show significant lipophilicity and water solubility, indicating its ability to penetrate the cell membranes and distribute properly in aqueous compartments, respectively. **ABQ-3** could possess an appropriate distribution volume and half-life at an acceptable dose and dose frequency related to its QPlogKhsa value. In particular, the penetration of a drug molecule from the blood into the brain is essential for the treatment of brain metastases of other cancer types. Both QikProp and SwissADME results showed that **ABQ-3** could cross this barrier easily. **ABQ-3** served a dug-likeness property, albeit to be out of the limit with a little rate for saturation in the bioavailability chart of SwissADME. **ABQ-3** was not a substrate of P-glycoprotein: a membrane protein that causes less drug concentration in the cell and triggers the development of resistance. **ABQ-3** matched the inhibition of CYP enzymes apart from CYP2D6, indicating that drug–drug interactions could emerge with molecules that undergo metabolism with these enzymes [72,73,74].

## 4. Materials and Methods

### 4.1. Chemistry

The synthetic experiments of **ABQ-3**, **ABQ-11**, and **ABQ-12** were carried out previously. Their structures were characterized by spectral analysis previously [23].

### 4.2. In Vitro Anticancer Screening

#### 4.2.1. NCI Single Dose Screening

The PQs were investigated for their growth inhibitory activity by the NCI (Bethesda, MD, USA) protocol at a 10 µM concentration in DMSO for 60 cancer cell lines. Compounds were added to the microtiter culture plates and incubated for 48 h at 37 °C. Sulforhodamine B (SRB) was applied for end-point detection. The percentage of the growth of the exposed cells was measured in comparison to the nonexposed control cells, and the findings of each tested compound were calculated [43,44,74].

#### 4.2.2. NCI Five-Dose Anticancer Screening

Serial 5 × 10-fold dilution was carried out from an initial DMSO stock solution before incubation at each individual concentration. The Developmental Therapeutics Program (DTP)-NCI screened the most effective compound (**ABQ-3**) for a higher testing level to determine GI_50_, TGI, and LC_50_ for each cell line after generating a dose–response curve, including concentrations of 0.01, 0.1, 1, 10, and 100 µM for **ABQ-3** [44,45,75].

#### 4.2.3. Cell Culture, Drug Treatment and MTT Assay

HCT-116 cells (provided by the RIKEN BRC through the National Bio-Resource Project of the MEXT/AMED, Japan (RCB2979)) and MCF-7 cells (Precision Bioservices, Frederic, MD, USA) were incubated in Dulbecco’s modified Eagle’s medium (DMEM) (Wako Pure Chemical Industries, Osaka, Japan) and RPMI 1640 (Wako Pure Chemical Industries, Osaka, Japan), respectively. All media (Wako Pure Chemical Industries) was supplemented with 10% fetal bovine serum (FBS) (Sigma Aldrich, St. Louis, MO, USA) and 89 μg/mL streptomycin (Meiji Seika Pharma, Tokyo, Japan) at 37 °C, 5% CO_2_ atmosphere. Both cells were cultured in a 24-well plate (Iwaki brand Asahi Glass Co., Chiba, Japan) at a 4 × 10^4^ cells/mL concentration for 48 h (the optimum cell number was quantified and linked with an earlier study) [40]. The stock solution of **ABQ-3** was prepared in DMSO (Wako Pure Chemical Industries, Osaka, Japan) and cisplatin in DMF (Wako Pure Chemical Industries, Osaka, Japan) at concentrations in the range of 0.1–10 mM and further was diluted with fresh culture medium. The concentration of DMSO and DMF in the final culture medium was 1% without affecting the cell viability [23,40].

MTT (Dojindo Molecular Technologies, Kumamoto, Japan) assay was used as previously explained [23,40] for the investigation of the effects of **ABQ-3** and cisplatin on cell viability. HCT-116 and MCF-7 cell lines were subjected to **ABQ-3** and cisplatin at 1, 3, 10, 30, and 100 μM concentrations at 37 °C for 48 h before being stained with an MTT solution and further incubated for 4 h. After the removal of supernatants, 100 μL DMSO and DMF (for cisplatin) were added to each well. For the analysis of the absorbance of the solution, a plate reader Infinite M1000 (Tecan, Mannedorf, Switzerland) was used. All experiments were applied with three repeats, and IC_50_ values were identified as the drug concentrations, which decreased the absorbance to 50% of the control values.

#### 4.2.4. Apoptosis Detection

HCT-116 and MCF-7 cells were incubated with **ABQ-3** and cisplatin at an IC_50_ concentration for 12 h before the cell death detection kit (PromoKine, Heidelberg, Germany) was carried out with some modifications to the manufacturer’s protocol [40]. Both cell lines were subjected to a binding buffer and staining solution and were then analyzed by fluorescence microscope Biorevo Fluorescence BZ-9000 (Keyence, Osaka, Japan) [76].

#### 4.2.5. Statistical Data

All findings were exhibited as means ± SD. Data were screened using a one-way analysis of variance, and diversity was accepted at * *p* < 0.05, ** *p* < 0.01, and *** *p* < 0.001. GraphPad Prism7 (GraphPad Software, San Diego, CA, USA) was used for the determination of the IC_50_ values.

### 4.3. In Silico Studies

#### 4.3.1. Molecular Docking

Initially, DNA was downloaded from RSCB (PDB IDs: 2GWA [54] and 2GB9 [55]) and was then prepared by PrepWizard of Maestro. Afterward, the docking grid was generated by the Grid generation of Maestro. **ABQ-3**, **ABQ-11**, and **ABQ-12** were also prepared using the LigPrep module of Maestro. Ultimately, Glide/XP docking protocols were administered for all these ligands [40,51,52,53,54,55].

#### 4.3.2. Absorption, Distribution, Metabolism, and Excretion (ADME) Estimation

The QikProp [57] and SwissADME web tool [58] were used for the determination of several important pharmacokinetic parameters of **ABQ-3**.

## 5. Conclusions

The quinone-based compound **ABQ-3** was selected for NCI-60 during in vitro screening at five doses towards a huge panel of cancer cells, including HCT-116 and MCF-7 cell lines. **ABQ-3** displayed 2.00 µM GI_50_, 4.37 µM TGI, and 9.55 µM LC_50_ values against HCT-116 cells, whereas these values were detected as 2.35 µM, 6.05 µM and 30.60 µM, respectively, against MCF-7 cells, demonstrating the growth inhibitory, cytostatic, and cytotoxic effects of **ABQ-3** on these cell lines. Based on MTT screening, this compound also showed significant cytotoxicity against HCT-116 and MCF-7 cells. Furthermore, the cell death assay demonstrated that **ABQ-3** enhanced apoptotic activity in HCT-116 and MCF-7 cell lines when compared with cisplatin. Molecular docking studies suggested the strong DNA binding of **ABQ-3**. In silico ADME prediction indicated that **ABQ-3** afforded positive drug-likeness values, thus, making this titled compound a potentially good and orally active anticancer agent for future studies.

## Figures and Tables

**Table 1:** The anticancer effects of **ABQ-3**, **ABQ-11**, and **ABQ-12** on cancer and healthy cells compared to imatinib.

Compound	Cell Type (IC_50_, μM)				
	K562 ^a^	Jurkat ^a^	MT-2 ^a^	PBMC ^a^	SI ^c^
**ABQ-3**	0.82 ± 0.07	1.51 ± 0.29	5.41± 0.95	8.37 ± 1.33	10.21
**ABQ-11**	0.28 ± 0.03	0.92 ± 0.08	2.50 ± 0.29	4.42 ± 1.18	15.78
**ABQ-12**	0.98 ± 0.22	1.99 ± 0.22	5.75 ± 0.81	8.49 ± 1.75	8.66
Imatinib ^b^	6.02 ± 0.92	8.65 ± 1.35	19.30 ± 3.41	35.56 ± 2.95	5.90

^a^ Cell lines include K562, Jurkat and MT-2 leukemia cells and peripheral blood mononuclear cells (PBMCs) (healthy). ^b^ Used as a reference. ^c^ Selectivity Index (SI) = IC_50_ for PBMCs/IC_50_ for K562 cells.

**Table 2:** The GI_50_, TGI, and LC_50_ values of **ABQ-3**.

Molecule	**ABQ-3 (NCI: D-827196/1)**		
Panel/Cell Line	GI_50_ (µM)	TGI (µM)	LC_50_ (µM)
**Leukemia**			
CCRF-CEM	2.93	-	>100
HL-60(TB)	2.18	5.54	>100
K-562	2.58	9.59	>100
MOLT-4	2.88	-	>100
RPMI-8226	2.23	5.69	>100
SR	2.37	8.06	>100
**NSLC**			
A549/ATCC	21.70	>100	>100
EKVX	1.79	3.43	6.56
HOP-62	12.20	25.40	53.00
HOP-92	1.40	2.84	5.78
NCI-H226	12.30	27.60	62.10
NCI-H23	14.80	55.00	>100
NCI-H322M	33.30	>100	>100
NCI-H460	25.50	>100	>100
NCI-H522	13.30	29.50	65.30
**Colon Cancer**			
COLO 205	19.20	38.80	78.10
HCC-2998	16.80	31.70	59.90
HCT-116	2.00	4.37	9.55
HCT-15	13.70	27.90	57.00
HT29	23.50	54.10	>100
KM12	3.75	44.30	>100
SW-620	3.92	18.90	96.70
**CNS Cancer**			
SF-268	11.40	52.20	>100
SF-295	10.30	23.70	54.50
SF-539	15.50	29.40	55.80
SNB-19	10.30	22.10	47.40
SNB-75	4.49	19.10	45.30
U251	7.50	21.00	49.30
**Melanoma**			
LOX IMVI	1.79	3.59	7.18
MALME-3M	14.00	28.20	56.80
M14	12.00	26.60	59.20
MDA-MB-435	2.74	7.13	26.60
SK-MEL-2	11.00	24.10	52.80
SK-MEL-28	14.90	28.80	55.60
SK-MEL-5	6.45	19.10	44.30
UACC-257	1.86	3.96	8.45
UACC-62	10.60	22.70	48.70
**Ovarian Cancer**			
IGROV1	1.78	3.35	6.29
OVCAR-3	2.12	4.53	9.67
OVCAR-4	1.71	3.10	5.60
OVCAR-5	16.80	35.70	75.80
OVCAR-8	1.84	3.84	8.02
NCI/ADR-RES	2.89	9.87	79.60
SK-OV-3	10.80	22.70	47.80
**Renal Cancer**			
786-0	18.70	34.00	61.60
A498	18.00	47.20	>100
ACHN	3.94	13.60	36.90
CAKI-1	10.40	22.20	47.50
RXF 393	9.14	21.40	47.10
SN12C	12.70	26.30	54.40
TK-10	28.60	52.10	94.90
UO-31	11.10	23.10	48.00
**Prostate Cancer**			
PC-3	4.51	20.60	65.10
DU-145	25.20	69.80	>100
**Breast Cancer**			
MCF-7	2.35	6.05	30.60
MDA-MB-231/ATCC	1.99	4.49	10.50
HS 578T	17.40	72.40	>100
BT-549	13.30	26.60	53.40
T-47D	1.83	4.26	
MDA-MB-468	1.29	2.75	5.85

**Table 3:** The cytotoxicity of **ABQ-3** on breast cancer and CRC cells, based on MTT assay at five dose concentrations (1, 3, 10, 30, and 100 μM).

Compound	IC_50_ Value (µM)	
	MCF-7 Cell Line	HCT-116 Cell Line
**ABQ-3**	7.46 ± 2.76	5.22 ± 2.41
**Cisplatin**	17.65 ± 4.55	22.19 ± 5.29

**Table 4:** The docking score, glide gscore, and glide emodel findings of **ABQ-3**, **ABQ-11**, and **ABQ-12** (PDB ID: 2GWA and 2GB9).

Compound	2GWA			2GB9		
	Docking Score (kcal/mol)	Glide Score (kcal/mol)	Glide Emodel (kcal/mol)	Docking Score (kcal/mol)	Glide Score (kcal/mol)	Glide Emodel (kcal/mol)
**ABQ-3**	−4.980	−4.980	−48.650	−6.610	−6.610	−54.810
**ABQ-11**	−4.717	−4.717	−45.691	−6.441	−6.441	−50.710
**ABQ-12**	−4.739	−4.739	−44.709	−6.552	−6.552	−51.805

**Table 5:** Predicted pharmocokinetic properties of **ABQ-3**.

Compound	QPlogPo/w (−2 to +6.5)	QPlogS (−6.5 to +0.5)	QPlogKhsa (−1.5 to +1.5)	QPlogBB (−3 to +1.2)	CNS (−2.0 to +2)	Human Oral Absorption% (>80% is High, <25% is Poor)	Rule of Five	Rule of Three
**ABQ-3**	2.639	−4.260	−0.034	−0.232	0	100.000	0	0

## Data Availability

Not applicable.

## Sample Availability

Samples of the compounds **ABQ-3**, **ABQ-11**, and **ABQ-12** are available from the authors.
